# Solution structures in alkali nitrates and nitrites at high concentrations

**DOI:** 10.1039/d5ra07630g

**Published:** 2026-03-09

**Authors:** Sebastian T. Mergelsberg, Trent R. Graham, Emily T. Nienhuis, Hsiu-Wen Wang, Ashley R. Kennedy, Lawrence M. Anovitz, Jacob G. Reynolds, Robert G. Felsted, Charles T. Resch, Carolyn I. Pearce

**Affiliations:** a Pacific Northwest National Laboratory Richland WA USA sebastian.mergelsberg@pnnl.gov trent.graham@pnnl.gov; b Chemical Sciences Division, Oak Ridge National Laboratory Oak Ridge TN USA; c Central Plateau Cleanup Company Richland WA USA; d Department of Crop and Soil Sciences, Washington State University Pullman WA USA

## Abstract

In highly concentrated electrolyte solutions, where classical models often fail, specific ion–solvent interactions dictate bulk properties. Here, we combine small-angle X-ray scattering (SAXS) and Raman spectroscopy to link molecular coordination to mesoscale structure across alkali nitrate and nitrite solutions. Our results reveal a structural hierarchy driven by cation identity in that smaller cations (Li^+^, Na^+^) form discrete contact ion pairs, while larger cations (K^+^, Rb^+^) promote increasingly disordered local coordination and extended solute–solvent networks. Using nitrite salts as a structural control, we confirm this organizing principle across different anion geometries. Cs^+^ undergoes a concentration-induced transition to a uniquely ordered state, resulting in a highly structured solution. This work provides a direct, mechanistic explanation for how cation choice dictates the ‘rules’ of solution architecture, offering a predictive basis for understanding phase behavior in complex industrial and environmental systems.

## Abstract 2

SAXS and Raman reveal smaller cations favor localized ion pairs, while larger cations induce cooperative mesoscale structuring, resolving molecular–mesoscale interactions in electrolytes relevant to industrial, energy & waste management applications.

## Graphical abstract

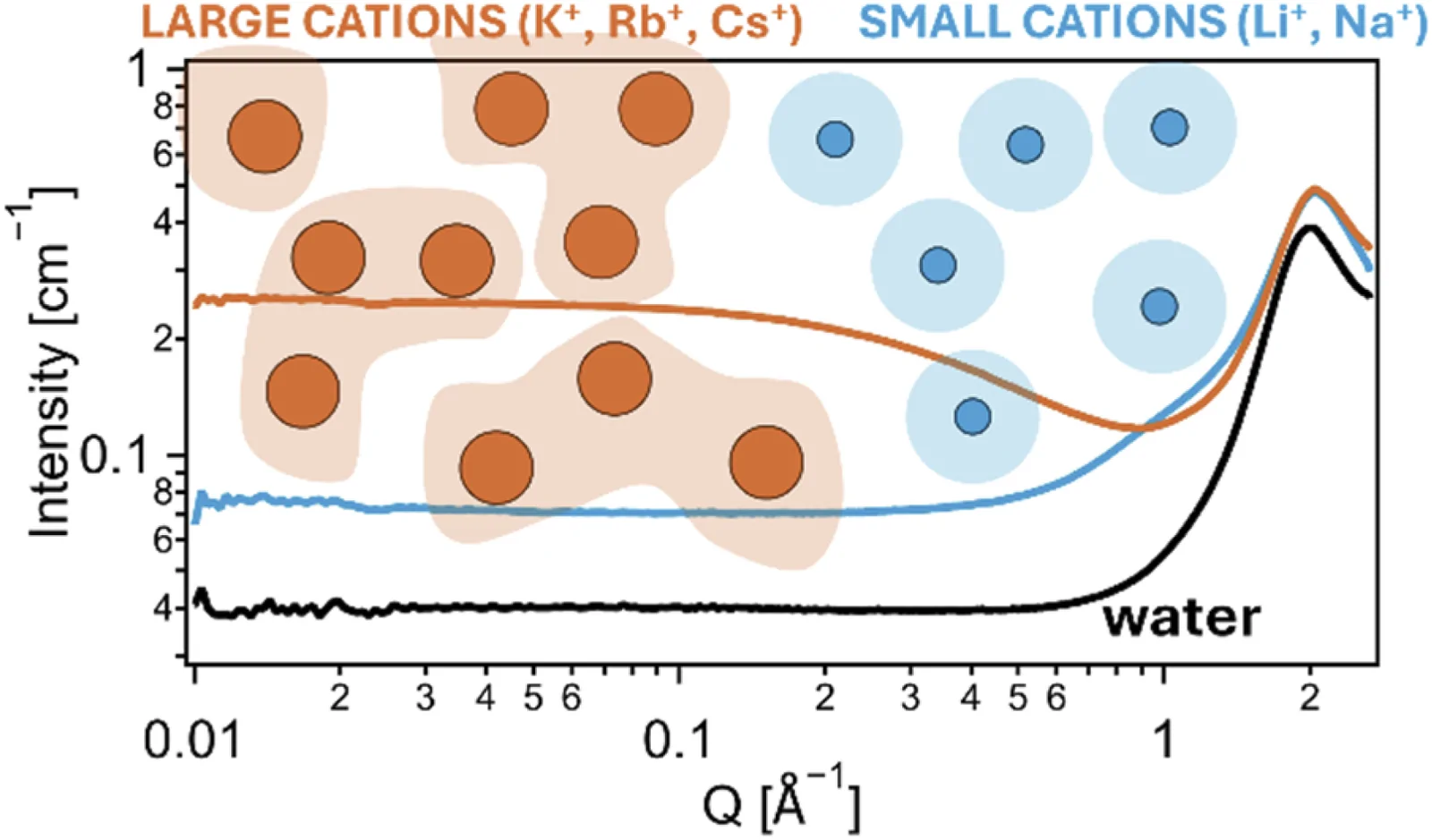

## 1. Introduction

The physicochemical properties of high-concentration electrolyte solutions are of interest to industrial processes, energy storage, and the management of concentrated chemical waste.^1–4^ These environments represent extreme chemical conditions in which models derived from dilute systems are insufficient to predict structure or reactivity. One example is the highly alkaline nuclear waste at the Hanford Site, where high concentrations, chemical heterogeneity, and prolonged exposure to radiation alter bulk properties that require molecular-level insight.

Alkali nitrate salts comprise the bulk of the dissolved electrolytes within this waste.^5,6^ In these water-limited solutions, the interplay between cation–solvent and cation–anion interactions becomes the dominant force controlling solution organization.^7,8^ Still, the identity of the counter-ion contributes critically to whether direct ion pairs or solvent-separated configurations prevail. This raises the central question of how the intrinsic properties of an alkali cation, namely its size and charge density, control the emergent, collective structure of the solution at the nanoscale. In aqueous solution, there is a fundamental size divide between Na^+^ and K^+^. Li^+^ and Na^+^ bind tightly to four to six waters, while K^+^, Rb^+^, and Cs^+^ accommodate six to ten waters in weaker, more variable hydration shells^9,10^ with the latter producing labile hydration shells and ps-scale fluctuations in solvation.^11–13^

Although the influence of cation identity on bulk properties is generally appreciated,^14–16^ a direct mechanistic link to nanometer-scale architecture remains poorly defined.^17^ Solution speciation reflects a dynamic equilibrium between contact ion pairs (CIP), solvent-separated ion pairs (SSIP), and free ions,^18^ and we postulate that these species yield distinct mesoscale structures reflective of solvent–solvent interactions.^19,20^

Anion geometry also modulates solution structure. The nitrate anion is trigonal planar with its single charge delocalized across three oxygens, resulting in a diffuse hydration shell and weak water interactions.^21,22^ By contrast, the nitrite anion is bent (C_2v_ symmetry) and polar, distributing charge over only two oxygens. This geometry enhances ion–water interactions and is reflected in the higher viscosity of nitrite solutions.^23^ Nitrate–nitrite thus provides a useful pair for testing how anion geometry perturbs cation-driven architectures.

Despite decades of study, the evidence for ion pairing and clustering in alkali nitrates remains inconsistent. Smaller cations such as Li^+^ and Na^+^ remain predominantly solvated at low concentration, with contact and solvent-separated ion pairs emerging only at higher molality.^24,25^ K^+^ and Rb^+^ form ion pairs more readily, although these associations are thermodynamically weaker.^26,27^ Both NaNO_3_ and KNO_3_ have been reported to form extended clusters,^28–30^ while Cs^+^ appears anomalous, with neutron diffraction suggesting little clustering under comparable conditions.^31^ Taken together, these mixed results underscore the need for systematic comparison across the series which we address here.

To resolve these inconsistencies, we hypothesize that low charge-density electrolytes such as K, Rb, and Cs nitrate form disordered, fluctuating ion networks at the mesoscale. Increasing cation or anion charge density destabilizes these networks through repulsive interactions, giving rise to more structured short-range order in the form of SSIPs and CIPs. Thus, we expect periodic structures to emerge for Li and Na nitrate, especially at higher concentrations, with nitrites and chlorides amplifying this effect. To test this hypothesis, we investigated the aqueous alkali nitrate series from dilute to saturation and extended the study to Na^+^ and K^+^ nitrite solutions as structural perturbations. Using small-angle X-ray scattering (SAXS) to resolve mesoscale architecture and Raman spectroscopy to probe local coordination, we relate solvation environment to solution structure, showing how cation identity governs concentrated electrolytes.

## 2. Experimental methods

### 2.1 Solution preparation

Solutions containing NaNO_3_ (Sigma Aldrich, ≥99%), LiNO_3_ (Sigma Aldrich, 99%), RbNO_3_ (Alfa Aesar, 99.8% trace metals basis), CsNO_3_ (Alfa Aesar, 99.8% trace metals basis), KNO_3_ (Fisher Chemicals, 99%), NaNO_2_ (Sigma-Aldrich, ≥97%) and KNO_2_ (Sigma-Aldrich, ≥96%) were evaluated. Solutions were generated by weighing out solids by mass into a bottle, then diluting by mass with MilliQ water. All solution mixtures were made in a N_2_ filled glovebox and allowed to sit undisturbed for seven days before being syringe filtered with 0.45 µm SFCA filters (Thermo Scientific, Nalgene). Concentrations of the most saturated solutions are reported in Table S1. These solution concentrations were measured *via* ICP-OES due to the presence of undissolved solids prior to analysis. The concentrations for the more dilute samples were determined based upon the amount of reagent added to the known amount of water and were not further analyzed for precise concentrations.

### 2.2 ICP-OES

Aliquots of each sample were taken to determine cation concentration *via* inductively coupled plasma optical emission spectroscopy (ICP-OES) using a PerkinElmer Optima 2100 DV ICP-OES with an AS93 auto sampler. The following 1000 µg mL^−1^ standards were used to calibrate the instrument: K (Ultra Scientific), Na (Inorganic Ventures), Li (Inorganic Ventures), Rb (Inorganic Ventures), Cs (Inorganic Ventures). Each standard (0.8 mL) was diluted with 196 mL 1.4% double distilled nitric acid (Fisher Chemical, Optima grade). Each sample was diluted with 1.4% double distilled nitric acid in a range between 20 k and 200 k using Fisher Elite pipettes before a Helix Tracey 4300 DV spray chamber and SeaSpray nebulizer were used to disperse the sample for analysis at a flow rate of 1.5 mL min^−1^.

### 2.3 Small angle X-ray scattering

SAXS data was acquired at beamline 12-ID-C of the APS7 using a constant incident photon energy of 20 k eV (*λ* = 0.6199 Å) to measure samples in transmission through a 1.5 mm ID silica capillary (Charles Supper SiO2 capillaries, 10 µm wall thickness). The beam center and detector configuration were calibrated using a silver behenate standard. For each sample, data was captured for 30 exposures of 1 s each at a sample-to-detector distance of 2.188 m, from *q* = 0.008 to 1 Å^−1^. The water and solutions were pumped into the capillary using an automatic sampler and constantly agitated in the capillary to avoid heating or settling of the sample. All data processing was completed using Irena 2.728 in IgorPro 9.02 (WaveMetrics, Inc., Lake Oswego, OR, USA). Data were normalized using in-line photodiodes and scaled by measuring water at identical conditions to the samples. Samples were then averaged for each composition. The averaged background scattering patterns of the silica capillary were then subtracted from all samples. All data were fit using the unified scattering model and one peak function to account for the pre-peak scattering.

### 2.4 Raman spectroscopy

Raman spectroscopy was performed on a Horiba LabRAM HR spectrometer with a Nikon Ti-E inverted microscope. A 632.81 nm continuous laser light source was focused through a 40× microscope objective. Spectra were collected between 100–4000 cm^−1^. Spectra were obtained by averaging 10 scans with 30 s exposures per spectral region. Post acquisition processing included derivative-based detection and replacement of cosmic ray outliers across replicate scans, arithmetic averaging, and Lorentzian peak fitting by least-squares minimization.

Raman spectroscopy of the solid salts was performed using a Horiba LabRAM Odyssey Raman spectrometer with a 100 mW 532 nm laser (Cobolt 08-DPL) and a 100 mW 785 nm laser (Horiba IPS H-Type laser), through a 10× magnification Olympus objective (numerical aperture of 0.25). The detector (Synapse EM) was a 1600 element CCD array with a gain of 400, used after an 1800 lines per mm diffraction grating. The confocal pinhole was set to 100 µm. Spectra were collected between Raman shifts of 60 and 4000 cm^−1^ using the 532 nm laser to collect a full spectrum of the nitrate vibrational modes, and spectra were collected between 1030 and 1100 cm^−1^ using the 785 nm laser to collect high resolution spectra of the *v*_1_ symmetric stretch. Spectral baselines were removed using an 8th order polynomial fit to regions without spectral intensity. The Raman spectra were collected using the LabSpec 6 software program, and the Raman data were plotted using the Origin 2025 software program.

## 3. Results and discussion

### 3.1 SAXS resolves anion-dependent solution structures for aqueous sodium salts

**Fig. 1:**
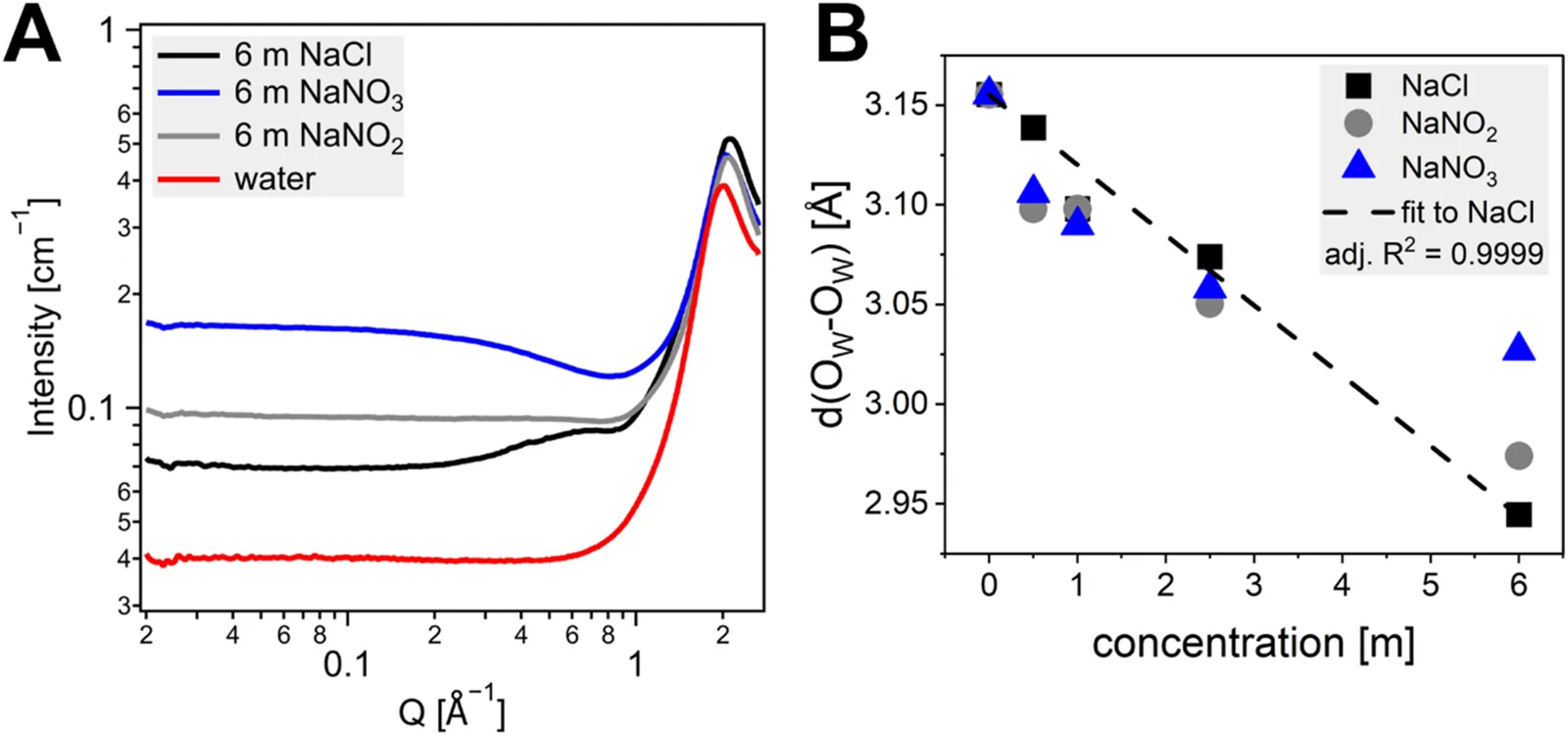
Fundamental structural differences between sodium salt solutions. (A) SAXS patterns of 6 m NaCl, NaNO_2_, and NaNO_3_ solutions, highlighting the unique low *q* region sensitive to mesoscale ordering in the nitrate and nitrite systems, which is absent in the NaCl solution. (B) Position of the water–water correlation peak as a function of salt concentration, demonstrating the near-ideal linear behavior of NaCl in contrast to the non-linear solvent structure changes induced by NaNO_2_ and NaNO_3_. The dashed black line is a linear fit to the NaCl data. The uncertainty of d(O_w_–O_w_) is within the symbol size.

To establish a baseline for the structural role of the nitrate anion, solutions of NaCl, NaNO_3_, and NaNO_2_ at concentration of 6 m (molality; mole solute per kg water solvent) were measured using small-angle X-ray scattering (SAXS) at identical measurement conditions (Fig. 1A). Note that the use of molality rather than molarity (M, mole solute per L solution) ensures that comparisons are made at equal solute-to-solvent mole ratios. Features observed in the three SAXS patterns are a direct reflection of anion identity and size effects, both relating to ion–ion/ion–water interactions, electronic contrast density, and solute volume fraction. In Fig. 1A, the investigated *q*-range provides quantitative information from the atomic up to the nanometer scales. At high-*q* values, above 0.8 Å^−1^ (2π/*q* < ∼8 Å), interatomic scattering is observed, with one dominant peak at *q* ≈ 2 Å^−1^, corresponding to the mean water–oxygen (O_w_) distance between neighboring water molecules. At intermediate-*q* values, between 0.1 and 0.7 Å^−1^ (2π/*q* ≈ 63–8 Å), a broad correlation peak at *q* ≈ 0.6 Å^−1^ is observed in NaCl solution but is not present for the other two solutions. The observation of a broad correlation peak (also known as the SAXS pre-peak), has been reported for chloride salt solutions of monovalent (NaCl), divalent (MgCl_2_, CaCl_2_, SrCl_2_, BaCl_2_), and trivalent (ErCl_3_) metal cations at single digit molality concentrations.^32,33^ These pre-peak signals, in general, scale with the electronic densities, with multivalent cations produce considerably clear pre-peak features as shown by SrCl_2_ and ErCl_3_ solutions at 3 m.^32^ Corresponding simulations (on di- and trivalent chloride salt solutions) have decoded that SAXS pre-peak is a collective structural correlation effects, meaning that some pairwise atom–atom structure factors are positive and some are negative, and the scattering pre-peak depends on how these contributions interfere, both destructively and constructively.

The molecular species of a solvated cation (viewed as one molecular entity) contributes mostly to the SAXS pre-peak signals, *i.e.*, the intermolecular cation⋯O_w_, O_w_⋯O_w_, and cation⋯cation correlations between two different solvated cation entities separated by some intermediate distances (∼8–11 Å). Although the hydration shell structure around Na^+^ is more flexible and a faster water exchange in Na^+^ hydration shell^34^ can be expected due to a smaller hydration free energy than that of di- or trivalent cations discussed here,^34^ we believe that the same molecular origination can be used to explain the observed pre-peak in 6 m NaCl solution (Fig. 1A). In contrast to NaCl, in the mid-*q* region, NaNO_3_ solution displays a kink, reaching to a plateau with decreasing *q*, whereas NaNO_2_ solution shows only a vaguely visible kink/plateau pattern. As will be shown in the later discussion, NaNO_2_ solutions at lower concentrations (2.5 m and below) display a more visible kink/plateau feature when compared to the 6 m solution data shown in Fig. 1A. The absence of SAXS pre-peak could indicate: (i) a coincident/almost exact cancelation between the positive and negative correlations; or (ii) the absence of any ordered structural correlations among various molecular entities/complexes that could formed in a solution. We associate the observed kink/plateau feature as salt-in-water nanoemulsion-like distribution,^35^ due to its similarity to the SAXS pattern observed for water-rich microemulsion^36^ systems in which nanoscale dispersions of two immiscible fluids (water and oil) are spatially isolated and stabilized by a monomolecular interface or a hydrotropic co-solvent. This salt-in-water nanoemulsion-like distribution reflects electron density fluctuations by thermal motion of solute and water molecules. The location of the kink at *q* = 0.3–0.4 Å^−1^ (2π/*q* ≈ 16–21 Å) corresponds to the spatial extent of electron-dense heterogeneities and could relate to intermediate-range interactions/attractions among ion–ion and ion–water. At low-*q*, below 0.1 Å^−1^, all three solutions show flat intensities. The lack of rising intensity at low-*q* suggests that large electron-dense “domains” of tens of nanometers are not present in these 6 m salt solutions. The stark difference between the NaCl, NaNO_3_ and NaNO_2_ solutions directly reflects the anion-specific effects on the intermediate-range solution structure. Results suggest the non-spherical geometry of NO_2_^−^ and NO_3_^−^ has a structural effect on the surrounding solvent beyond the length scales of a hydration sphere.

The measurements also demonstrate a fundamentally different correlation between salt concentration and the water solvent scattering peak for the three chemical systems. In the NaCl system, the water peak position scales linearly with concentration (Fig. 1B), with the measurements shown in Fig. S1. This strong linear trend (*R*^2^ = 0.9999) is expected for near-ideal binary mixtures. For the NaNO_2_ and NaNO_3_ solutions the effect of electrolyte concentration on the solvent peak position is non-linear. Up to 3 m concentrations, both solutions lead to a contraction of solvent structure relative to NaCl. At 6 m concentrations, the NaNO_2_ solutions are more like NaCl than NaNO_3_, leading to a 10% contraction in O_w_⋯O_w_ distances compared to pure water. This suggests that in the NaNO_3_ case, the solvent distances appear to saturate at some concentration, consistent with the micro-emulsion interpretation. At some concentration, the interface between solute and solvent no longer scales with concentration directly. In NaNO_2_ solutions, the highest concentrations approach the NaCl trend, possibly indicating only limited formation of micro-emulsion domains.

### 3.2 Cation-dependence of the nitrate solution structures

**Fig. 2:**
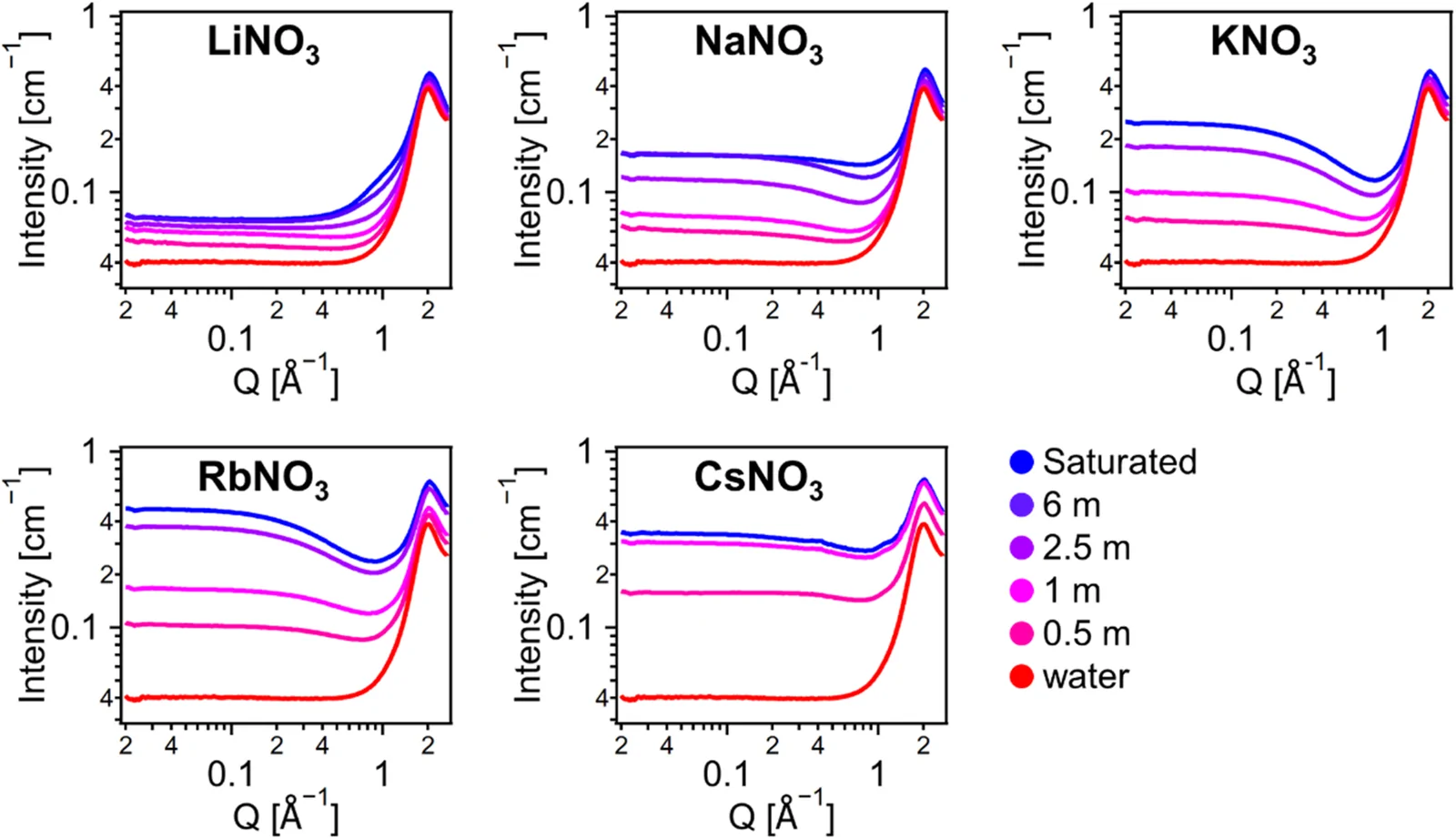
Concentration-dependent solution structure for the full alkali nitrate series. SAXS patterns of all five salts (LiNO_3_, NaNO_3_, KNO_3_, RbNO_3_, and CsNO_3_) and the corresponding changes from dilute to saturated conditions, resulting in the distinct concentrated solution structures summarized in Fig. 3. Exact concentrations for the saturated conditions are provided in Table S1.

To determine how cation identity influences this nitrate-driven ordering, we next measured the SAXS patterns of the full alkali metal nitrate solution series, with the highest concentrations controlled by the solubility of each nitrate salt (Table S1). The complete concentration-dependent SAXS data for each salt are presented in Fig. 2. This data visually shows how distinct scattering features evolve from the dilute solution state as concentration increases.

Since the solubility of alkali metal nitrates at 25 °C decreases in the order of LiNO_3_ (12.81 m) > NaNO_3_ (10.79 m) > RbNO_3_ (4.79 m) > KNO_3_ (3.53 m) > CsNO_3_ (1.4 m),^37^ the 0.5 and 1 m SAXS data provide a full comparison crossing the solution series.^38^ Up to 1 m, the kink/plateau feature in the mid-*q* region can be seen in all five solutions, but is progressively more visible with increasing the electron density and total number of electrons on the cation. Note that, at concentrations at/below 1 m, the lightest alkali cation, Li^+^, produces a weak kink/plateau scattering, which is only visible after subtraction of pure water scattering from the reduced data (Fig. S2). The CsNO_3_ SAXS data at saturation (1.4 m; blue curve in Fig. 2, CsNO_3_ panel) shows evidence of diffraction-like peaks. These diffraction peaks (labelled in Fig S3) do not coincide with any nitrate salts or polymer substances used in this experiment, but are close to the *d*-spacings previously reported for large low-symmetry complexes, such as gas hydrates,^39^ alkali cluster ions,^40^ and solutions at high pressures.^41^ However, none of these phases are likely to form in the solution composition and condition studied here, and thus these diffraction-like peaks remain unidentified.

**Fig. 3:**
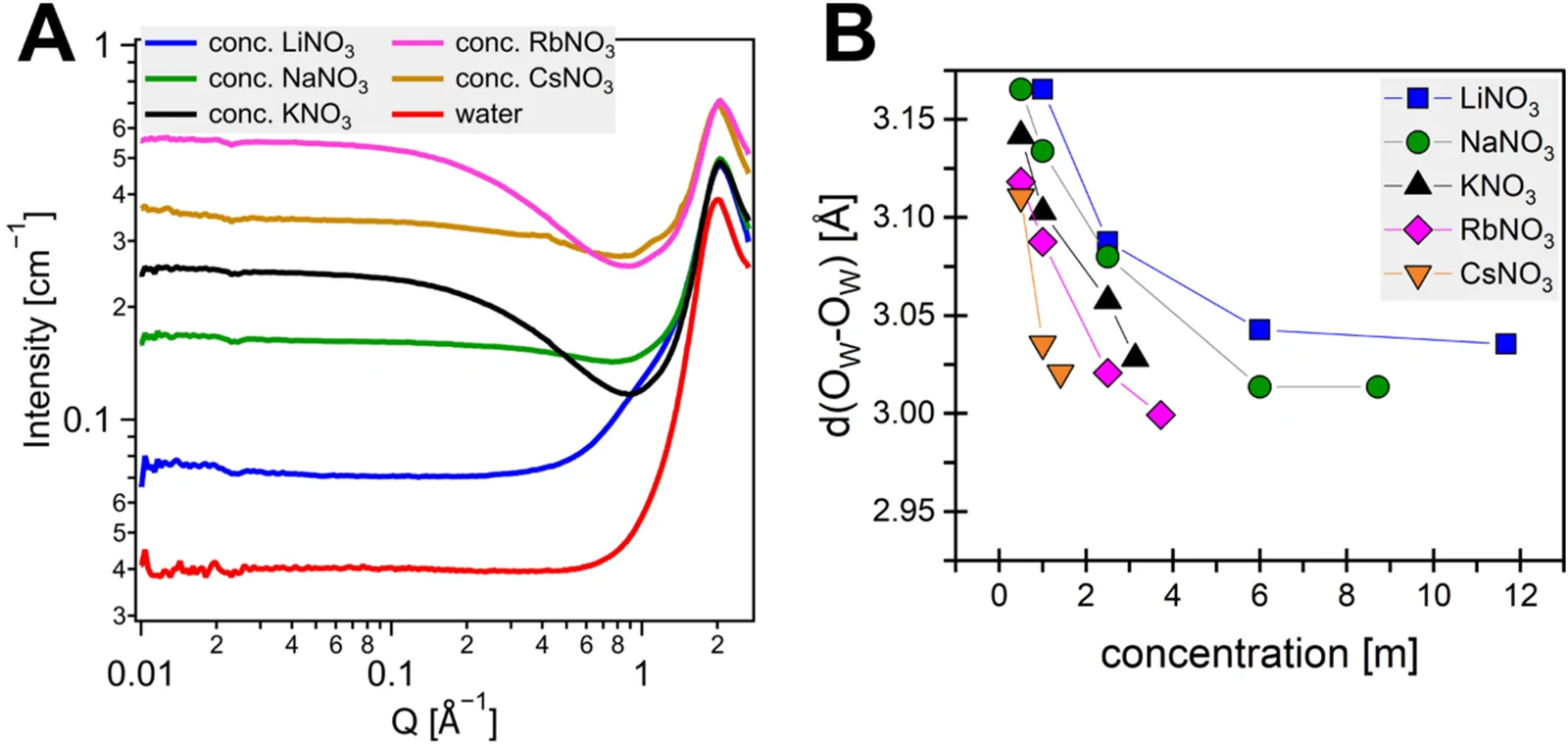
Cation identity dictates the final mesoscale order and solvent network at high concentration. (A) Comparison of SAXS patterns from saturated or near-saturated alkali nitrate solutions, summarizing the three distinct structural classes that emerge from the data in Fig. 2. (B) Position of the water–water correlation peak as a function of molality, showing the strong, non-linear effect of all alkali nitrate salts on the solvent network. The uncertainty of d(O_w_–O_w_) is within the symbol size.

At 2.5 m concentration and up close to the solubility limits in KNO_3_ and RbNO_3_ solutions (at 3.1 and 3.7 m, respectively), the kink/plateau intensities can be clearly observed in the KNO_3_ and RbNO_3_ concentration series (Fig. 2, KNO_3_ and RbNO_3_ panels). By comparison, a similar evolution in kink/plateau also exist in the LiNO_3_ and NaNO_3_ concentration series, but a pre-peak hump starts to show up in the high-*q* (approximately *q* ≈ 1 Å^−1^) at concentrations above 2.5 m. At 6 m and close to saturation in LiNO_3_ and NaNO_3_ solutions (at 11.7 and 8.7 m, respectively), the disappearance of kink/plateau at the mid-*q* may be masked by a broad pre-peak growing in at *q* ≈ 1 Å^−1^, and intensities at high-*q* stop to increase with salt concentrations (Fig. 2, LiNO_3_ and NaNO_3_ panels). In both the LiNO_3_ and NaNO_3_ concentration series, the scattering from nanoemulsion-like distribution at low concentrations transitions to the pre-peak development with increasing concentrations (above 2.5 m). If we assume the appearance of pre-peak is related to the development of specific structure factors (*i.e.*, similar to the origin of pre-peak in chloride salt solutions), the observed scattering transitions indicate a change of solution structure from density fluctuations to formation of correlated molecular motifs at the highest concentrations (in LiNO_3_ and NaNO_3_ solution system). Thus, analysis of the solutions at close to their solubility limits, summarized in Fig. 3A, shows cation-specific effects that can be grouped into two apparent structural evolution classes, with smaller cations (Li^+^ and Na^+^) promoting development of specific structure factors (dominated by pairwise molecular scattering), and nitrate solutions of larger cations (K^+^, Rb^+^ and Cs^+^) preserving greater electron density fluctuations.

To constrain the lengthscale of the low-*q* nano-emulsion features, we use a small-angle scattering mode commonly used to model random density fluctuations to fit the data. For each system, we only used the strongest scattering signal of the microemulsion feature, which is the 1 m solution for LiNO_3_, 6 m for NaNO_3_, and the saturated conditions for KNO_3_, RbNO_3_, and CsNO_3_. The first model is a general correlation length model,^42^ where the scattering intensity *I* as a function of momentum transfer *q* is defined as:1
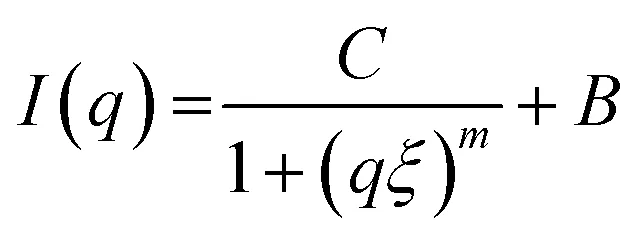


Dependent upon the scalars *C* and *B*, as well as the correlation length *ξ* and the Lorentz exponent *m*, indicative of the scatterer fractal dimension. For the most concentrated systems that exhibit these scattering features, the derived correlation lengths are 2.01(3) for LiNO_3_, 1.27 (1) Å for NaNO_3_, 2.10 (3) Å for KNO_3_, 2.31(2) Å for RbNO_3_, and 1.35(9) Å for CsNO_3_. The corresponding Lorentz exponents are 2.22(5), 1.77(2), 1.92(4), 1.80(2), and 1.75(8). An example fit is shown in Fig. S4. For fits to LiNO_3_ data, only the water-subtracted data were used (Fig. S2). These results suggest that the scattering features are of a consistent fractal dimension (1.8 to 2.2) similar to that found in disordered organic molecules.^43^ The length scales measured by this model represent a lower bound and suggest very small/highly fluctuated electron-dense regions. The correlation length values are quite small, which implies the electron-dense clusters are coupled to ion/molecular-scale phenomena and highly fluctuating with a broad size distribution. The presence of small pre-peaks to the main water peak at ∼1 Å^−1^ implies that there is some weak nano-scale correlation between these electron-dense clusters, especially for K^+^, Rb^+^, and Cs^+^ (Fig. 2 and 3). Presence of these peaks also implies there are longer-range correlations not captured by the nano-emulsion features. The relative trends in sizes as a function of cation suggests that the electron-dense region is correlated with the cation and not the anion or solvent molecules. The features occur solution at distinct length scales for each cation. A precise physical interpretation of this scattering feature is currently not possible and will require further analysis of the scattering patterns of concentrated nitrate solutions and discrete molecular models of these conditions.

The effect of the mesoscale solute structure on the solvent–solvent distances approximately follows the non-linear trend established for NaNO_3_ above, with the caveat that larger ions have a stronger concentration-dependent effect (Fig. 3B). Li and Na reach the highest solubilities in our measurements and show relatively little change in the measured distances between the two highest concentrations. The dependence of this distance on K, Rb, and Cs concentration is very strong, quickly reaching values of ∼3.05 Å at concentrations < 4 m. Note that no distances below 3 Å are observed, which indicates that there likely is a structural limit at which the distance between water molecules becomes too short and the salt precipitates. Along similar lines, for the highly soluble salts (NaNO_3_ and LiNO_3_), the solvent–solvent distance reaches a minimum value that is cation-specific. This plateau is also observed in the scattering patterns (Fig. 2). Between the 6 m and the saturated condition, the low-*q* tail (*q* < 0.1 Å^−1^) does not change intensity and only the peak at *q* ≈ 1 Å^−1^ grows. This background intensity is related to the mean electron density of the solution.^32^ At the same time, the correlations between hydrated cation molecular motifs that make up the pre-peak feature continue to increase. Thus, SAXS effectively demonstrates that cation identity dictates the mesoscale structure of nitrate solutions. These correlations do not resolve the molecular-level mechanisms that drive this trend. Thus, we turn to Raman spectroscopy to resolve the underlying molecular arrangements corresponding to the trends in solution structure.

### 3.3. Raman spectroscopy measures local coordination environments

Raman spectroscopy probes the NO_3_^−^ and NO_2_^−^ anions' local coordination environments. Given that Raman has been used to characterize alkali nitrate solutions for at least 5 decades,^18,44–49^ the primary contribution of the Raman data in this manuscript is that the data corresponds to concentrations of solutions measured in SAXS. For NO_3_^−^ solutions, we focused our analysis on the trends in two key non-degenerate vibrational modes, the *v*_1_ symmetric stretch (1050 cm^−1^) and the *v*_4_ in-plane bend (720 cm^−1^). We first measured the solids (Fig. S5 and S6) to demonstrate these modes do not bear resemblance to the anisotropic bonding environment of the solids. Both *v*_1_ and *v*_4_ modes are sensitive to perturbations of the anion's symmetry and their peak positions and line shapes serve as complementary and robust reporters for the degree of cation–anion association. The nitrate bands are known to be composed of overlapping contributions from multiple species (free ions, solvent-SSIPs, CIPs).^7,44–49^ These shifts have been attributed to ion-pairs because they approach the wavenumber of the given salt as the concentration increases.^18^ Rudolph *et al.* confirmed, using density functional theory, that the Raman spectra shifts correspond to ion-pairs for Na^+^–NO_3_^−^.^50^ We also confirmed the absence of lattice vibrations at lower wavenumbers (Fig. S7 and S8), indicating no solid is present in these solutions.

**Fig. 4:**
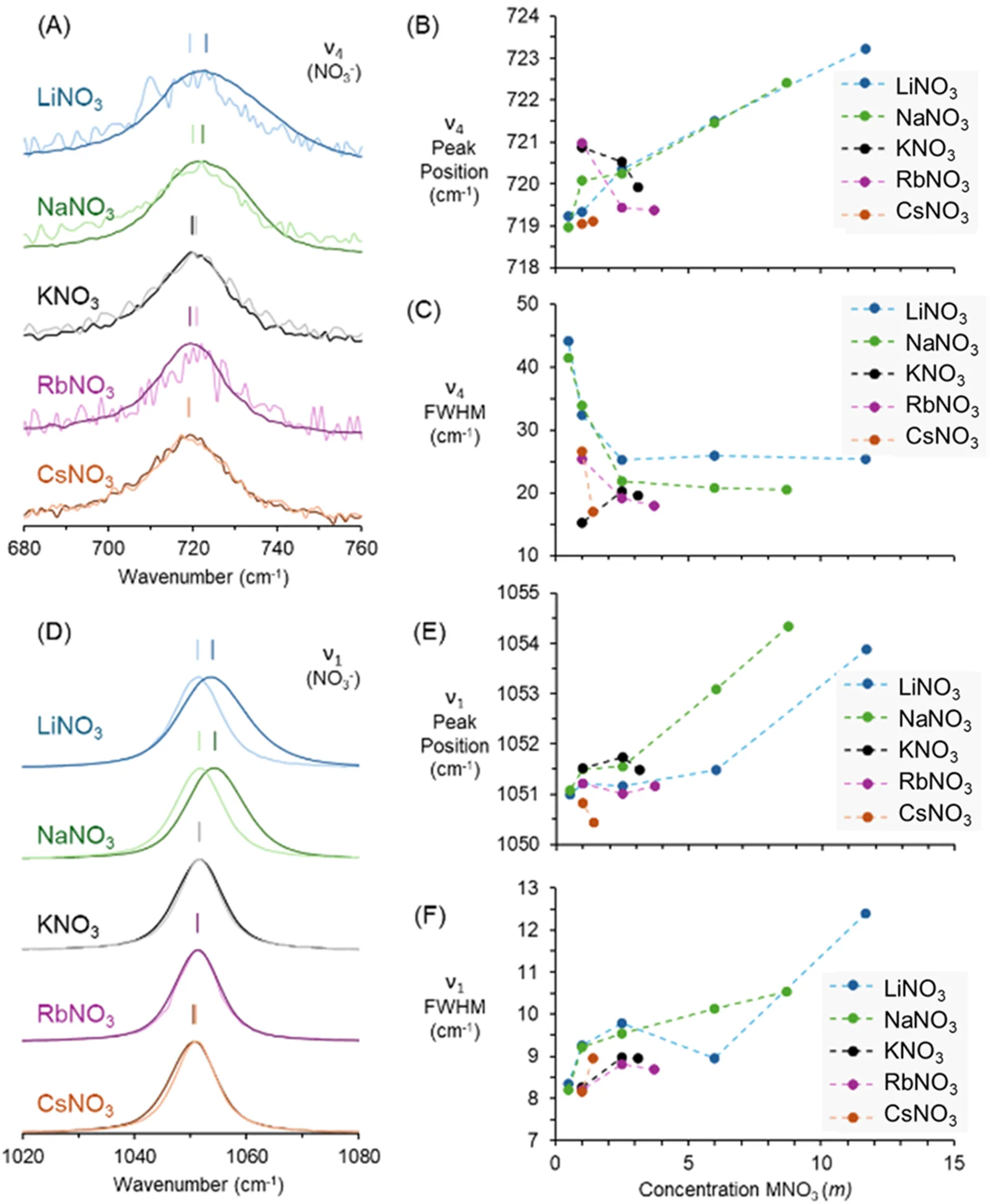
Cation-specific coordination trends in the *v*_4_ and *v*_1_ vibrational modes of NO_3_. (A) Stacked spectra of the *v*_4_ in-plane bend for each alkali nitrate, from 1 m (light lines) to saturation (dark lines). (B) Corresponding *v*_4_ peak positions as a function of molality. (C) FWHM of the *v*_4_ peak *versus* molality. (D) Stacked spectra of the *v*_1_ symmetric stretch. (E) Corresponding *v*_1_ peak positions as a function of molality. (F) FWHM of the *v*_1_ peak *versus* molality. Vertical marks over the spectra are provided to emphasize shifts in peak positions between concentrations.

The severe overlap of nitrate bands in alkali systems makes unique deconvolution challenging. Therefore, we track the overall peak position (first moment) and full width at half maximum (FWHM) as indicators of the average change in the coordination environment across the solution. The Raman results are shown in Fig. 4. Example fits (Fig. S9) and a table of line shape parameters (Table S2) are provided in the SI.

The Raman data reveal a hierarchy of interaction strength. For LiNO_3_ and NaNO_3_, both the *v*_1_ and *v*_4_ modes exhibit a pronounced and systematic blue shift with increasing concentration (Fig. 4B–E) with asymmetry of the band at high concentrations. This blue shift is a well-established spectroscopic signature that indicates a shift in the solution equilibrium towards contact ion pair (CIP) formation, where specific coordination, for example by high-charge-density Li^+^ and Na^+^ cations, perturbs the anion's symmetry.^18^ Furthermore, the subtle asymmetry that develops in the *v*_4_ band at the highest concentrations (Fig. 4A) suggests a distribution of local environments, consistent with a dynamic equilibrium between CIPs and solvent-separated species. Note that this is more subtle than signatures of ion pairing in divalent cation systems where new bands are resolved at elevated concentrations.^51,52^ Note that the SAXS data indicated solution ordering started in the LiNO_3_ system at 2.5 m whereas the *v*_1_ peak position did not start increasing until above 6 m. This likely indicates that SSIP or other longer-range features are responsible for the ordering observed in the SAXS data between 2.5 and 6 m LiNO_3_.

The *v*_1_ and *v*_4_ peak positions for KNO_3_ and RbNO_3_ remain fairly invariant across concentration, albeit with some difference between the most dilute conditions characterized (0.5 and 1 m). This could indicate that the insensitivity of band position at higher concentrations for these larger, lower-charge-density cations favor solvent-shared or solvent-separated species rather than contact ion pairs (CIPs). This appears inconsistent with the Law of Matching Water Affinities, which predicts that weakly hydrated anions such as nitrate should form CIPs with larger, weakly hydrated cations.^53,54^ An alternative hypothesis is that CIPs involving K^+^ and Rb^+^ do form, but their diffuse interactions perturb nitrate symmetry too weakly to produce Raman shifts, rendering them spectroscopically silent.^55^ The SAXS results discussed above favor the second interpretation, as the observed electron-dense regions are consistent with CIP formation and mesoscale ordering that may weaken short-range interactions. Likewise, the Raman shifts for all alkali salts approaches the value of the solid salts of the same alkali, which also supports the assumption of contact ion-pair formation.^18^

The FWHM for these salts remains broader than for Cs (Fig. 4C–F), suggesting a more isotropic distribution of local solvent environments rather than a single, highly ordered state. This predominance of cation-mediated interactions is consistent with cation-specific, mesoscale correlation features observed in their corresponding SAXS patterns.

Solutions of CsNO_3_ exhibit a third, unique behavior. Both the *v*_1_ and *v*_4_ modes display a distinct red shift upon increasing concentration. Such red shifts, which reflect a reduction in the effective N–O bond force constant. There is a significant narrowing of the peak FWHM to values approaching that of the crystalline solid,^56^ this points to a unique, concentration-induced transition into a highly ordered mode of association. This behavior may arise from the weak hydration and high polarizability of Cs,^57^ potentially enabling specific cation–anion or even cation–cation correlations that differ fundamentally from the other alkalis. These features emerge from an initially disordered coordination environment and provide a mechanistic basis for the distinct SAXS pre-peaks and structured patterns observed for CsNO_3_. Recent work on mixed hydroxide–nitrite solutions shows a similar trend. Cs^+^ disrupts ideal mixing and drives mesoscale segregation into Cs-rich domains, accompanied by preferential Cs^+^–NO_2_^−^ association.^58^ These findings reinforce that cesium uniquely promotes ordered, ion-specific architectures distinct from lighter alkalis.

### 3.4. Parallels to nitrite solutions

**Fig. 5:**
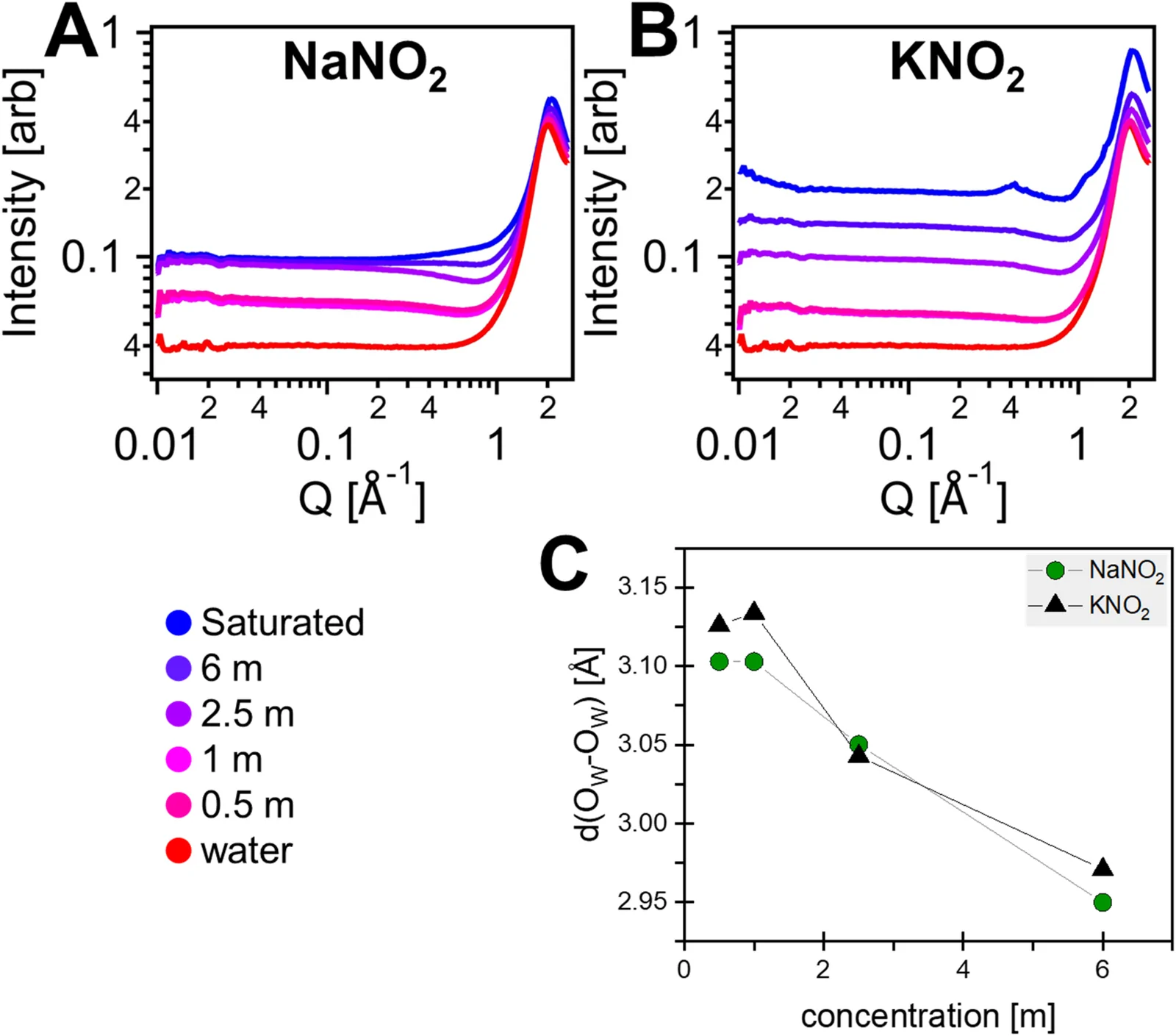
Concentration-dependent solution structure for Na and K nitrite. SAXS patterns of all (A) NaNO_2_ and (B) KNO_2_ as a function of concentration. (C) Position of the water–water correlation peak of the nitrite solutions as a function of molality, showing near-identical effects of the two salts on the solvent structure.

To further deconstruct the interplay between cation identity and anion geometry, we expanded our measurements to solutions of NaNO_2_ and KNO_2_. This comparison provides information to the extent of principles established for the nitrate series to a system with a different anion geometry. SAXS measurements (Fig. 5) demonstrate distinct solution scattering for nitrites compared to the nitrates. Both NaNO_2_ and KNO_2_ exhibit the complex scattering curve as presented in the NaNO_3_ system at concentrations < 6 m. In the case of NaNO_2_, the growth of the broad peak occurs at a lower concentration, above ∼2.5 m. Just like the NaNO_3_ system, growth of the broad peak also causes the low *q* background of the SAXS signal to become concentration independent. For KNO_2_, the scattering curve is largely congruent between concentrations, with a continuous increase in the background scattering. At the highest concentration of 22.4 m, the KNO_2_ data shows significant scattering peaks in the SAXS patterns, consistent with extensive ordering in a highly concentrated system. For both CsNO_3_ and KNO_2_ these extensive ordering peaks do not coincide with any lattice vibration in the Raman spectra (Fig. S7 and S8). At concentrations below 2.5 m, where both NaNO_2_ and KNO_2_ show similar solution structures, the impact on the solvent structure is only slightly different (Fig. 5C). This effect approximately follow trends in cation size, which suggests cation and solvent ordering are largely comparable at the mesoscale between the two systems at low concentrations. The effect on solvent structure 2.5 and 6 m is similar between NaNO_2_ and KNO_2_, suggesting a shift in the mesoscale structuring that decouples the cation size dependence from the solvent structuring. Thus, we predict Raman spectroscopy to reveal diverging nitrite trends between Na and K, assuming the anion is in two distinct configurations to enable similar mesoscale structures for cations of different sizes.

**Fig. 6:**
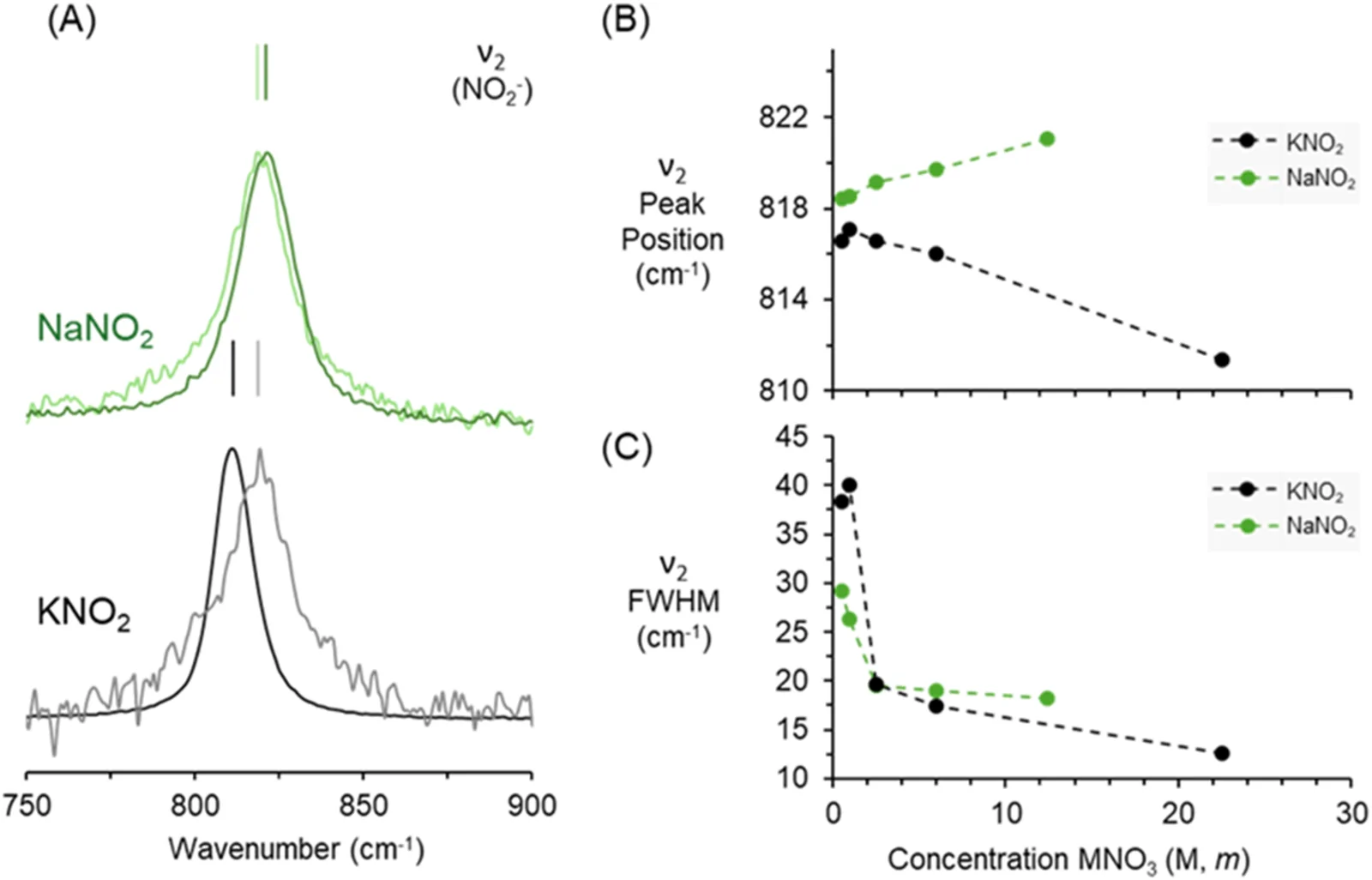
Raman spectroscopy reveals the perturbation of the water network in alkali nitrite solutions. Stacked spectra of the (A) *v*_2_ nitrite band alongside the (B) peak position and (C) the FWHM. In the stacked spectra, 1 m corresponds with the light lines and those at the highest respective concentration with the dark lines. Vertical marks over the spectra are provided to emphasize shifts in peak positions between concentrations.

For nitrite, we focus on the *v*_2_ bending mode (∼815 cm^−1^) because it is spectrally well separated from overlapping vibrational features. The Raman spectra of the nitrite *v*_2_ bending mode (815 cm^−1^) for both NaNO_2_ and KNO_2_ (Fig. 6) reveal that the overarching cation-driven principle holds, but with some anion-specific deviations. Example fits are shown in Fig. S10 with results for the series tabulated in Table S3. For NaNO_2_, the *v*_2_ peak exhibits a systematic blue shift (2 cm^−1^). This is consistent with the nitrate series and confirms that the equilibrium for Na^+^ is dominated by contact ion pair formation regardless of anion geometry. In stark contrast, the *v*_2_ peak for KNO_2_ undergoes a significant red shift (5 cm^−1^), a behavior markedly different from the stability observed in KNO_3_ solutions. This, coupled with the extreme and continuous narrowing of the FWHM to a final value of ∼12.5 cm^−1^, signals a concentration–driven transition into a uniquely homogeneous and highly ordered local environment, consistent with the pre-peak development observed in SAXS (Fig. S3). This suggests that the “permissive” K^+^ cation, by virtue of its larger size and weaker hydration, accesses a different distribution of coordination sites relative to the nitrite anion center. This enables the bent, dipolar nitrite anion to self-organize into a structure that is unavailable to the planar nitrate anion.

Synthesizing these observations reinforces our thesis that the cation dictates solution architecture through its size and hydration properties, while the anion moderates it. Larger cations promote greater local disorder and mesoscale heterogeneity, with ordering transitions emerging at high concentrations (*e.g.*, KNO_2_, CsNO_3_). The formation of discrete contact ion pairs by sodium is the overriding principle, confirmed by the consistent blue shift in the Raman spectra for both NaNO_3_ and NaNO_2_. Conversely, potassium acts as a permissive network-enabler for both anions. However, the nature of the network is anion-dependent. For nitrate, it is a heterogeneous, fluctuating system. For nitrite, the anomalous red shift and extreme peak sharpening in the Raman spectrum provide direct evidence of a transition to a uniquely ordered and cooperative structure, which provides a clear mechanistic basis for the exceptionally high solubility of KNO_2_.

## 4. Conclusion

The combination of SAXS and Raman spectroscopy provides a multi-scale picture of solution architecture in concentrated alkali nitrate and nitrite solutions. SAXS reveals that these systems segregate into distinct structural classes, ranging from near-ideal binary mixtures (LiNO_3_) to those with extensive inhomogeneities (most other nitrates and nitrites). The solvent structure is strongly dependent on cation radius and strongly non-linear for all nitrates. Raman spectroscopy resolves the anion-specific short-range order underlying this phenomenon. Both Li^+^ and Na^+^ are dominated by specific CIP coordination configurations. In contrast, the larger cations (K^+^, Rb^+^) enable NO_3_^−^ and NO_2_^−^ to form extended, cooperative solute–solvent networks, a process confirmed by the activation of otherwise weak or forbidden vibrational modes. The unique behavior of Cs^+^ leads to a third, highly structured state.

Together, these results establish a clear framework in which the coordination environment dictated by cation size is the definitive driver of nanometer-scale solution organization. Small, high-charge-density cations (Li^+^, Na^+^) enforce localized contact ion pairs, while larger cations (K^+^, Rb^+^) permit extended, cooperative solute–solvent networks through access to different coordination site distributions around the anion. Our investigation of the nitrite system confirms that this principle provides a mechanistic basis for understanding how the interplay between cations and anions dictates solution properties in the non-ideal, highly concentrated regime.

## Conflicts of interest

The authors declare no conflicts of interest.

## Supplementary Material

RA-016-D5RA07630G-s001

RA-016-D5RA07630G-s002

## Data Availability

The data supporting this article have been included as part of the supplementary information (SI). Supplementary information: tables of compositions, Raman fit parameters for alkali nitrate and nitrite solutions, SAXS datasets and analyses, and example fits of Raman spectra of alkali nitrate and nitrite solutions. See DOI: https://doi.org/10.1039/d5ra07630g.
