# Supplementary material for: Solution structures in alkali nitrates and nitrites at high concentrations
Source: RSC Adv. 2026 Mar 10;16(15):13347–57. doi: 10.1039/d5ra07630g (PMC12973280; doi:10.1039/d5ra07630g)
Supplement: RA-016-D5RA07630G-s002 [file RA-016-D5RA07630G-s002.pdf]

Supplementary Information (SI) for RSC Advances.
This journal is © The Royal Society of Chemistry 2026

SUPPLEMENTARY INFORMATION FOR:

Solution Structures in Alkali Nitrates and Nitrites at High Concentrations

Sebastian T. Mergelsberg[1*], Trent R. Graham[1*], Emily T. Nienhuis[1], Hsiu-Wen Wang[2], Ashley R. Kennedy[1], Lawrence M. Anovitz[2], Jacob G. Reynolds[3], Robert G. Felsted[1], Charles T. Resch[1], Carolyn I. Pearce[1,4]

[1]Pacific Northwest National Laboratory, Richland, WA, USA
[2]Chemical Sciences Division, Oak Ridge National Laboratory, Oak Ridge, TN, USA
[3]Central Plateau Cleanup Company, Richland, WA, USA
[4]Department of Crop and Soil Sciences, Washington State University, Pullman, WA, USA

S1. Supplementary Tables

Table S1. Measured concentrations, densities, and viscosities of select solutions.

Sample Name	Concentration target	Concentration actual	Density (g/cm^3)	Dynamic Viscosity (mPa*s)	Kinematic Viscosity (mm^2/s)
Sat. KNO_3	3.53 m K	3.13 m K	1.14270	0.994	0.870
Sat. $NaNO_3$	10.79 m Na	8.72 m Na	1.37485	2.830	2.059
Sat. $LiNO_3$	12.81 m Li	11.67 m Li	1.30391	3.330	2.554
Sat. $RbNO_3$	4.79 m Rb	3.72 m Rb	1.26390	0.968	0.766
Sat. $CsNO_3$	1.40 m Cs	1.41 m Cs	1.12916	0.939	0.832
Sat. $NaNO_2$	12.4 m Na	---	---	---	---
Sat. KNO_2	22.5 m K	---	---	---	---

Table S2. Parameters of the fit to the Raman spectra of alkali nitrate solutions. Peak position and FWHM are detailed.

Composition	Peak Position (cm-1)	FWHM (cm-1)	Peak Position (cm-1)	FWHM (cm-1)	Peak Position (cm-1)	FWHM (cm-1)
Conc. LiNO3	723.2	25.3	1053.9	12.4	1407.0	87.6
6 m LiNO3	721.5	25.9	1051.5	9.0	1398.4	107.4
2.5 m LiNO3	720.4	25.3	1051.2	9.8	1398.8	71.6
1 m LiNO3	719.3	32.4	1051.2	9.3	1400.7	55.9
0.5 m LiNO3	719.2	44.1	1051.0	8.4	1393.8	57.8
Conc. NaNO3	722.4	20.5	1054.3	10.5	1398.6	69.9
6 m NaNO3	721.5	20.8	1053.1	10.1	1398.6	69.9
2.5 m NaNO3	720.2	21.9	1051.6	9.5	1411.3	43.3
1 m NaNO3	720.1	33.8	1051.5	9.2	1394.5	69.6
0.5 m NaNO3	719.0	41.5	1051.1	8.2	1388.9	75.8
Conc. KNO3	719.9	19.5	1051.5	9.0	1396.4	71.2
2.5 m KNO3	720.5	20.2	1051.7	9.0	1396.5	71.8
1 m KNO3	720.9	15.2	1051.5	8.3	1395.6	50.6
0.5 m KNO3	719.4	40.9	1050.6	9.1	1392.1	66.0
Conc. CsNO3	719.1	17.0	1050.4	9.0	1395.4	55.0
1 m CsNO3	719.1	26.6	1050.8	8.2	1394.9	57.0
Conc. RbNO3	719.4	17.9	1051.2	8.7	1395.0	73.4
2.5 m RbNO3	719.4	19.1	1051.0	8.8	1394.3	69.7
1 m RbNO3	721.0	25.4	1051.2	8.2	1394.4	60.9

Table S3. Parameters of the fit to the Raman spectra of alkali nitrite solutions. Peak position and FWHM are detailed.

Composition	Peak Position (cm-1)	FWHM (cm-1)	Peak Position (cm-1)	FWHM (cm-1)	Peak Position (cm-1)	FWHM (cm-1)
Conc. KNO2	811.37	12.57	1241.17	58.64	1330.4	38.32
6 m KNO2	816.01	17.37	1232.6	66.35	1334.3	48.39
2.5 m KNO2	816.55	19.58	1230.44	69.11	1334.2	49.77
1 m KNO2	818.6025029	25.877	1227.18	71.49	1334	50.29
0.5 m KNO2	816.58	25.9	1222.75	76.89	1334	51.33
Conc. NaNO2	821.07	18.15	1242.62	64.36	1336.1	43.63
6 m NaNO2	819.73	19.01	1237.35	68.52	1335.3	46.61
2.5 m NaNO2	819.13	19.52	1232.92	71.62	1334.9	48.67
1 m NaNO2	818.5638417	26.301	1230.81	71.1	1334.5	49.25
0.5 m NaNO2	818.0415155	24.094	1226.8	32.82	1335.4	48.98

S2. Supplementary Figures

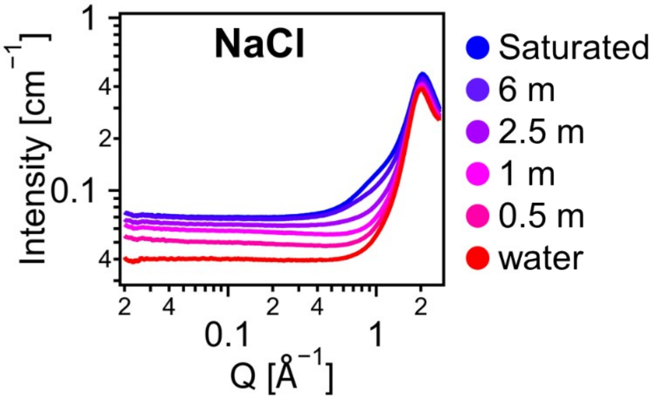

Figure S1. Concentration-dependent SAXS patterns for NaCl solutions.

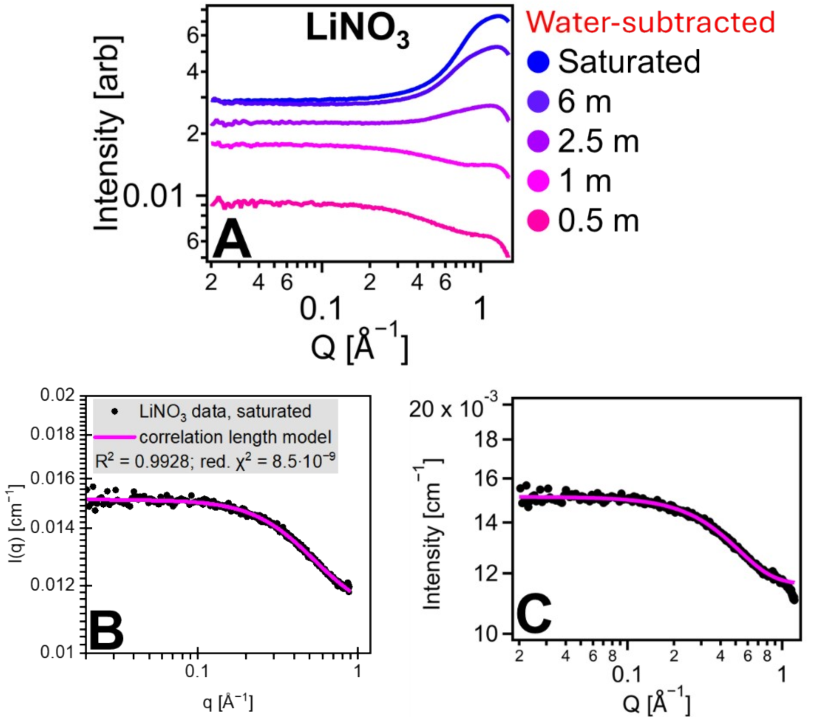

Figure S2. Water-subtracted Li nitrate patterns show peak feature only grows in at concentrations of 2.5 m and above. Using a two-peak fit to account for the broadening and shifting of the water peak, we can fit the peak at q ~ 1 Å^{-1}. This fit yields peak positions of 0.71 Å^{-1} for 2.5 m solutions, 0.95 Å^{-1} for 6 m solutions and 1.09 Å^{-1} for saturated solutions. This is equivalent to d-spacings of 8.82, 6.61, and 5.76 Å. The 1 m sample was fit using the correlation length model (B) and the unified fitting approach (C) between 0.02 < q < 0.9 Å^{-1}.

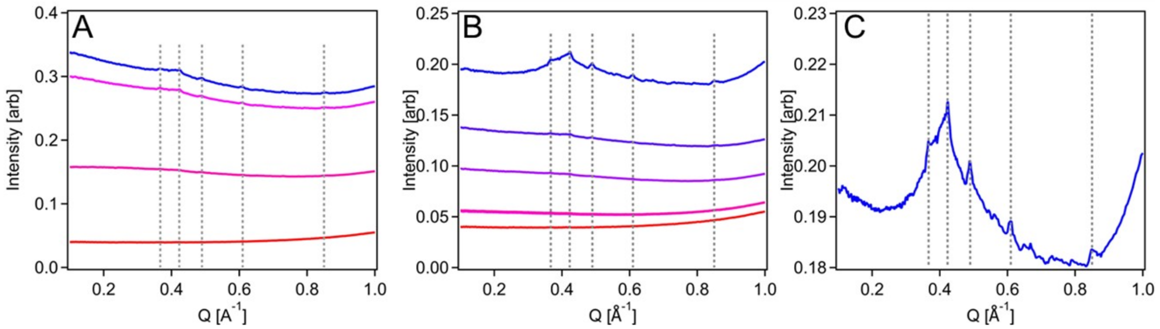

Figure S3. Emergence of concentration-dependent Bragg peaks at low q for $CsNO_3$ (A) and KNO_2 (B) systems. The saturated KNO_2 condition is highlighted in panel C, but peaks are first observed at 0.5 m for both systems. The grey lines indicate q = 0.367, 0.423, 0.490, 0.610, and 0.850 $Å^{-1}$, corresponding to d = 17.1, 14.9, 12.8, 10.3, and 7.4 Å, respectively.

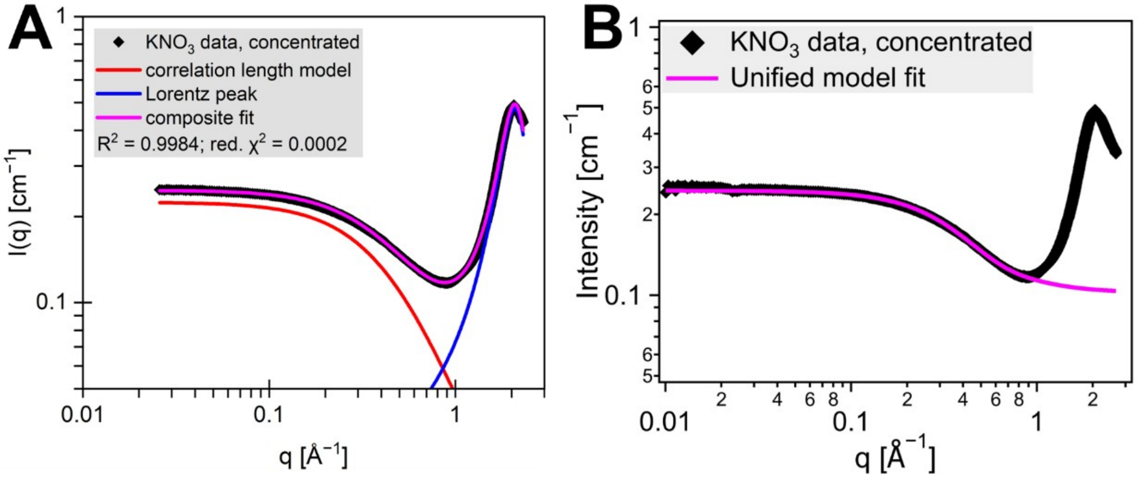

Figure S4. Example SAXS fits of both models to the concentrated KNO_3 solution data.

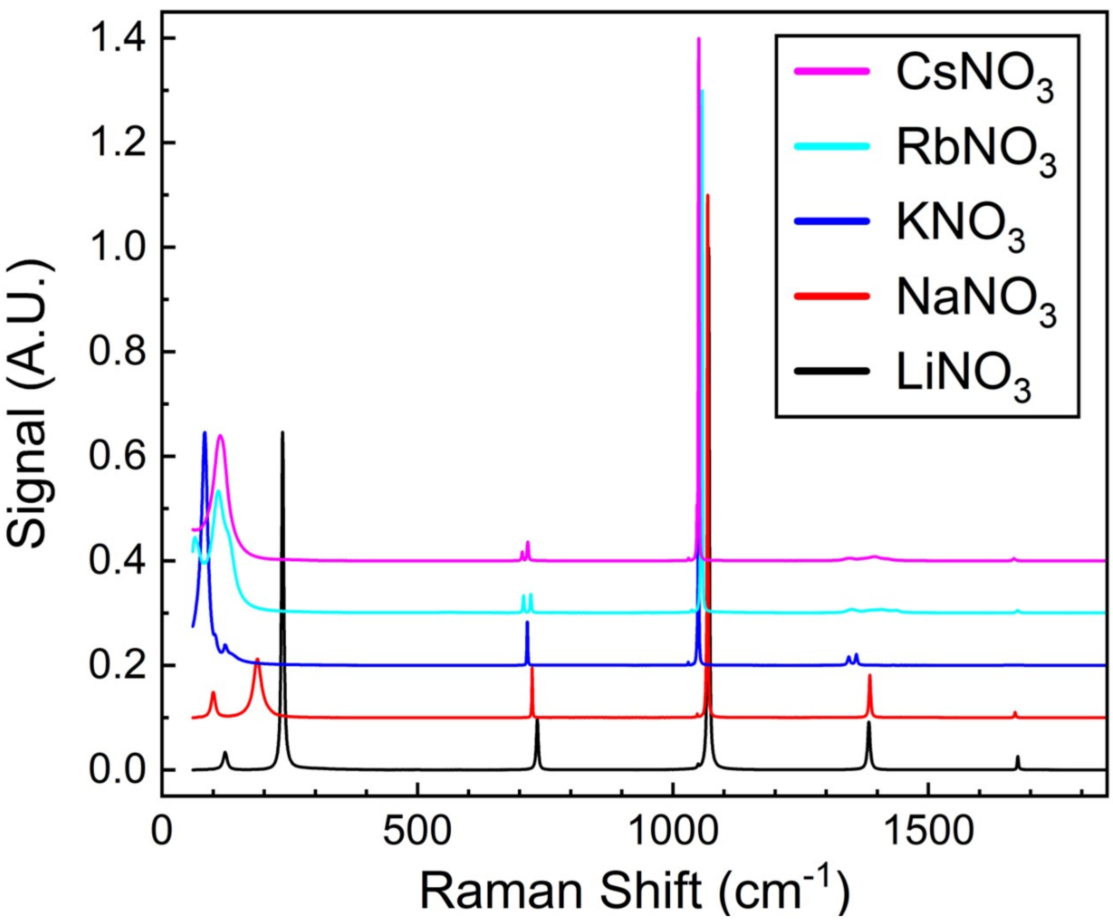

Figure S5. Raman spectra of the solid nitrate salts, showing characteristic peaks for each nitrate salt and the presence of lattice vibrations at low wavenumbers (< 500 cm^{-1}).

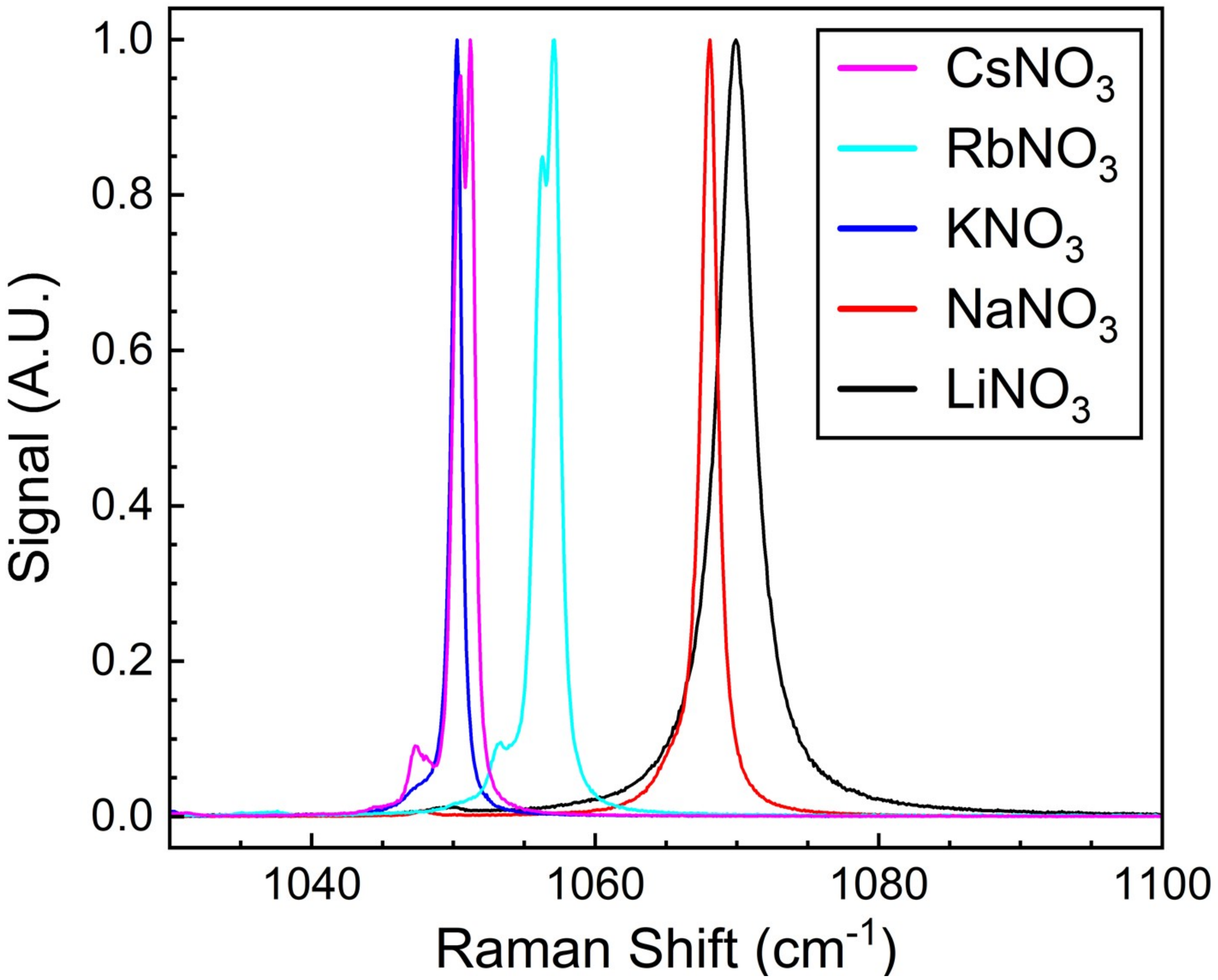

Figure S6. Raman spectra of the solid nitrate salts, showing additional detail of the strongest peak between 1030 and 1100 cm^{-1} for each salt. In the Raman data for the solutions, this peak is at ~1051 cm^{-1} at low concentration, shifting to 1055 cm^{-1} at higher concentrations. The structure-dependent peak anisotropies present in the spectra of the solid salts are not present for the equivalent peak of nitrate solutions (**Figure 4**).

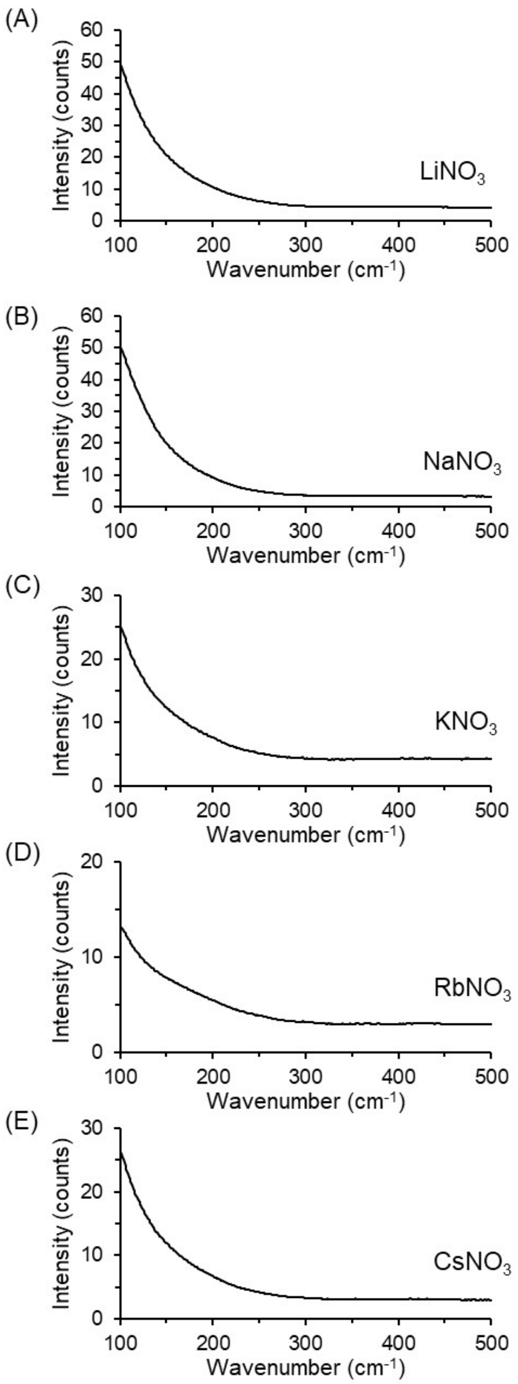

Figure S7. The Raman spectra of the saturated solutions of all nitrates. None of the solutions show any indication of lattice vibrations.

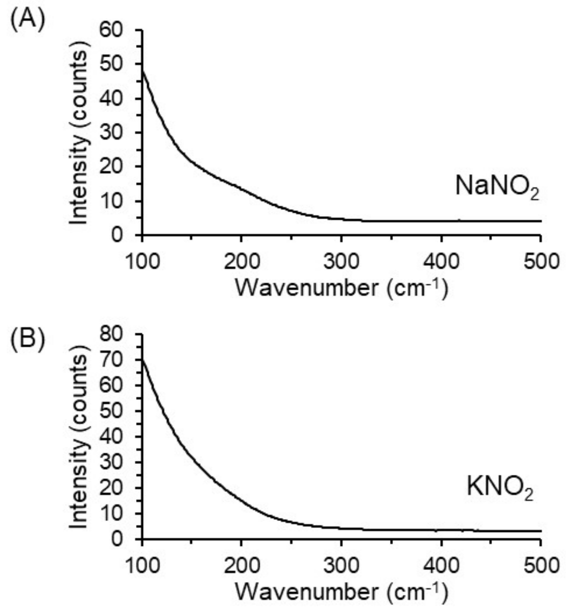

Figure S8. The Raman spectra of the saturated solutions of all nitrites. None of the solutions show any indication of lattice vibrations.

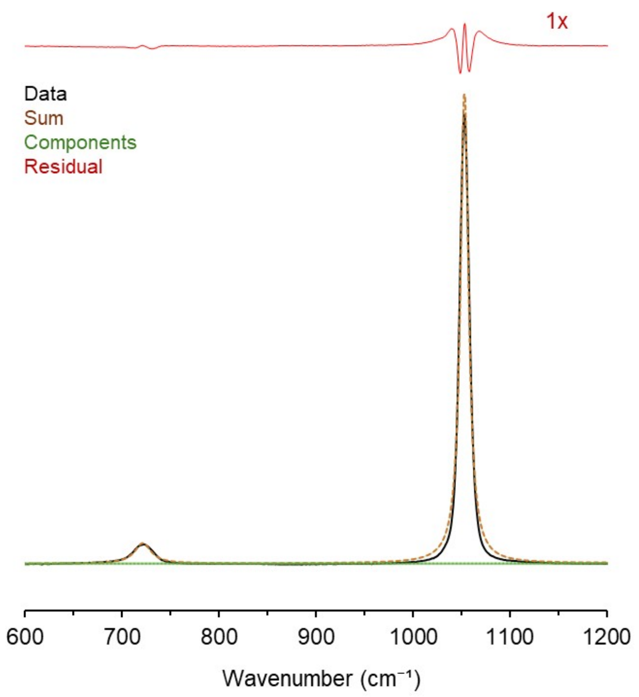

Figure S9. Example Raman fits for the 6 m $NaNO_3$ solution.

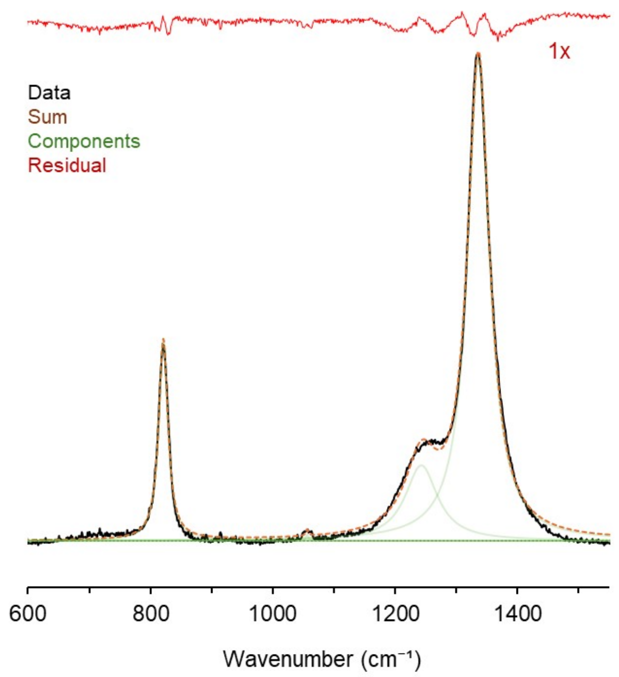

Figure S10. Example Raman fits for the saturated (12.4 m) $NaNO_2$ solution.
